# Integration and Potential Applications of Cardiovascular Computed Tomography in Cardio-Oncology

**DOI:** 10.1007/s11886-025-02206-x

**Published:** 2025-02-11

**Authors:** Muhammed Ibrahim Erbay, Venkat Sanjay Manubolu, Ashley F. Stein-Merlob, Maros Ferencik, Mamas A. Mamas, Juan Lopez-Mattei, Lauren A. Baldassarre, Matthew J. Budoff, Eric H. Yang

**Affiliations:** 1 Lundquist Institute at Harbor-UCLA Medical Center, Torrance, CA USA; 2 Department of Medicine, Istanbul Cerrahpasa University, Istanbul, Türkiye; 3 UCLA Cardio-Oncology Program, Division of Cardiology, Department of Medicine, University of California at Los Angeles, Los Angeles, USA; 4 Knight Cardiovascular Institute, Oregon Health and Science University, Portland, OR USA; 5 Keele Cardiovascular Research Group, Keele University, Keele, UK; 6 Lee Health Heart Institute, Fort Myers, FL USA; 7 Section of Cardiovascular Medicine, Yale School of Medicine, New Haven, CT USA

**Keywords:** Cardiovascular computed tomography, Cardio-oncology, Coronary artery calcium score, Risk stratification, Coronary artery disease

## Abstract

**Purpose of Review:**

Cardiovascular computed tomography (CCT) is a versatile, readily available, and non-invasive imaging tool with high-resolution capabilities in many cardiovascular diseases (CVD). Our review explains the increased risk of CVD among patients with cancer due to chemoradiotherapies, shared risk factors and cancer itself and explores the expanding role of CCT in the detection, surveillance, and management of numerous CVD among these patients.

**Recent Findings:**

Recent research has highlighted the versatility and enhanced resolution capabilities of CCT in assessing a wide range of cardiovascular diseases. Early detection of cardiac changes and monitoring of disease progression in asymptomatic patients with cancer may lessen the severity of CVD. It offers an essential means to assess for coronary artery disease when patients are either unable to safely undergo stress testing for ischemia evaluation or at risk of complications from invasive coronary angiography. Furthermore, CCT extends its utility to valvular diseases, cardiomyopathies, pericardial diseases, cardiac masses, and radiation-induced cardiovascular diseases, allowing for a comprehensive, noninvasive assessment of the entire spectrum of cancer treatment associated CVD. Looking to the future, the integration of artificial intelligence and machine learning algorithms holds potential for automated image interpretation, improved precision and earlier detection of subclinical cardiac deterioration, allowing opportunities for earlier intervention and disease prevention.

**Summary:**

CCT is a useful imaging modality for assessing the myriad cardiovascular manifestations of diseases such as coronary artery disease, cardiomyopathies, pericardial disesaes, cardiac masses and radiation-induced cardiovascular diseases. CCT has several advantages. Readily available non-cardiac chest CT scans of patients with cancer may help with improved cardiovascular care, enhanced ASCVD risk stratification and toxicity surveillance.

## Introduction

The field of cardio-oncology continues to grow in its significance as cardiovascular disease (CVD) is the top cause of morbidity and non-cancer mortality in the growing population of cancer survivors [1]. Increased cardiovascular risk is likely due to a combination of the effect of shared risk factors, cancer itself, and cancer therapy-related adverse effects. There are many underlying shared risk factors between CVD and cancer including hypertension, hyperlipidemia, obesity, physical inactivity, poor diet, diabetes, and smoking. Different therapies and certain cancer types may be more associated with atherosclerotic cardiovascular disease (ASCVD); however, traditional ASCVD risk models do not take into account this heterogeneity of cardiovascular (CV) risk [2]. In addition, The cardio-oncology population encompasses different groups of individuals, including those with many shared cardiovascular and oncologic risk factors, patients undergoing pre-treatment assessment before initiating cancer therapeutics, those actively receiving cancer treatment, and individuals who have completed therapy. This latter group includes patients who may still have cancer, as well as cancer survivors without current evidence of disease or recurrence, thus no longer classified as having active cancer. Computed tomography (CT) is readily used for cancer staging but can also reveal underlying CVD [3]. The use of cardiovascular imaging techniques, such as cardiovascular computed tomography (CCT), has emerged as a valuable tool for the evaluation and management of cardiovascular conditions in patients with cancer. CCT offers several advantages that make it a valuable imaging modality in cardio-oncology. These include non-invasive and high-resolution imaging of coronary arteries with measurement of coronary artery calcium (CAC) scores, evaluating for obstructive and nonobstructive plaque, differentiating between ischemic and nonischemic etiologists of cardiomyopathy etiology, and diagnosing pericardial disease, pulmonary embolism, and calcific valvular heart diseases. Our review aims to highlight the current indications, advantages, and challenges of the use of CCT in the field of cardio-oncology.

## Current Guidelines for Cardiovascular CT in Cardio-Oncology

Cardio-oncology guidelines recommend the use of CCT for chest pain evaluation and measurement of CAC for CV risk assessment (Table 1). The European Society of Cardiology (ESC) recommends the use of coronary computed tomography angiography (CCTA) to exclude acute coronary syndrome (ACS) in cancer-related Takotsubo syndrome as a Class 1 indication with C level of evidence (LOE) [4]. The ESC Cardio-oncology guidelines also recommend the use of CAC scoring to reclassify baseline CV risk in addition to traditional risk factors. (Class 1, LOE C) Furthermore, beginning at 5 years after chest radiotherapy, CCTA screening can be considered for high-risk patients to detect radiation-induced coronary artery disease (CAD) and valvular calcifications, and it can be used to guide the management of ischemia as a Class 1 indication with C LOE. It is important to note, that despite the guideline’s endorsement of these CCTA indications, the LOE is categorized as C (driven by expert opinion and/or low-level of evidence specifically in patients with cancer), highlighting the need for further research, such as randomized-controlled trials and prospective studies in the cardio-oncology population. A Society of Cardiovascular Computed Tomography (SCCT) expert consensus endorsed by IC-OS (International Cardio-Oncology Society) has published a statement that provides recommendations of applications of CCTA among patients with cancer, which include using readily available non-cardiac chest CT scans to report CAC absence or presence, and estimation of CAC extent in asymptomatic patients with cancer. Moreover, non-contrast gated CAC score CT is recommended for baseline CVD risk factor evaluation as a way to further refine ASCVD risk stratification to help guide decision-making to start lipid-lowering therapy, and prior to planned valvular interventions. Table 1 lists Class I and/or strong recommendations by the ESC and SCCT. (8) ACC CV Imaging and Cardio-oncology Councils have released a joint statement about the significance of using multimodal imaging in patients with cancer [5]. CCTA can assess CAD and cardiac masses as well as help with the preplanning of transcatheter valve repair procedures. Additionally, CCTA can evaluate for cardiotoxicity-caused ACS-like symptoms. For cancer survivors, CCTA can assess traditional ASCVD risk. The American College of Cardiology/American Heart Association (ACC/AHA) has not released an official joint expert consensus document, yet, due to the need for more rigorous evidence in the cardio-oncology population to guide recommendations.

**Table 1:** Recommended Use of Cardiovascular CT by current guidelines in Cardio-Oncology

Guidelines	Indications	Class	Level of Evidence
ESC^4^	Exclude acute coronary syndrome in cancer related Takotsubo syndrome	1	C
	Reclassification of CV risk using CAC scoring	1	C
	Assessment of radiation-induced cardiovascular diseases to guide ischemia management	1	C
SCCT expert consensus document endorsed by IC-OS^6^	Reporting on presence or absence of CAC on non-cardiac chest CT scans for cancer screening	Strong Recommendation	
	In asymptomatic patients with available non-cardiac chest CT scans, CAC scores should be used to improve ASCVD risk stratification	Strong Recommendation	
	Baseline evaluation for screening and optimizing underlying CVD risk factors	Strong Recommendation	
	If no previous noncardiac chest CT scans are available, CAC scan is recommended in all asymptomatic patients who are not under antilipidemics and with intermediate ASCVD risk 5–20% consistent with ACC/AHA, ESC, SCCT guidelines	Strong Recommendation	
	In asymptomatic patients with cancer being evaluated prior to chest irradiation, clinicians should review available non-cardiac chest CT reports and/or images and if there is evidence of CAC presence in a patient without history of ASCVD, to improve CV risk stratification and reduce ASCVD risk.	Strong Recommendation	
	In asymptomatic patients with cancer with a history of prior chest irradiation and no history of ASCVD, a CAC scan should be considered 5–10 years after the last RT for evaluation of radiation-induced CAD. If no evidence of ASCVD, it should be considered to repeat at 5–10-year intervals thereafter. Acquired images should be carefully evaluated for valvular and pericardial calcifications.	Strong Recommendation	
	CCT is recommended prior to planned valvular interventions (TAVR, TMVR, and TTVR) in patients with radiation-induced valve disease	Strong Recommendation	
	CCT can be used as an adjunct imaging modality in the evaluation of cardiac masses, often as a complimentary technique to other imaging modalities	Strong Recommendation	
	CCT should be considered in patients undergoing cardiac tumor resection to evaluate for anatomical relationships between tumor and coronary arteries for surgical planning, and to exclude obstructive CAD.	Strong Recommendation	
	CCT can be useful for evaluating pericardial fluid and characterizing it by measuring the CT attenuation value in Hounsfield Units. It can be useful for evaluating pericardial thickness and pericardial calcification in patients with cancer with suspected pericardial disease.	Moderate Recommendation	
ACC CV Imaging and Cardio-oncology Councils^5^	*Pre-cancer treatment*: CAD Assessment, Pericardial disease, Cardiac masses, Preplanning for transcatheter valve repair procedures *Cardiotoxicity assessment*: CAD Assessment, ACS-like symptom evaluation *Post cancer treatment*: CAD Assessment and Pericardial disease *Cancer survivorship*: Traditional ASCVD risk assessment and radiation sequalae		

CV: Cardiovascular disease, CAC: Coronary Artery Calcium

*Abbreviations* CV: Cardiovascular, CVD: Cardiovascular Disease, CAC: Coronary Artery Calcium, CT: Computed Tomography, ASCVD: Atherosclerotic Cardiovascular Diseases, RT: radiotherapy, TAVR/TMVR/TTVR: transcatheter aortic/mitral/tricuspid valve replacement

## Uses of Cardiovascular Computed Tomography (CCT) in Cardio-Oncology

**Fig. 1:**
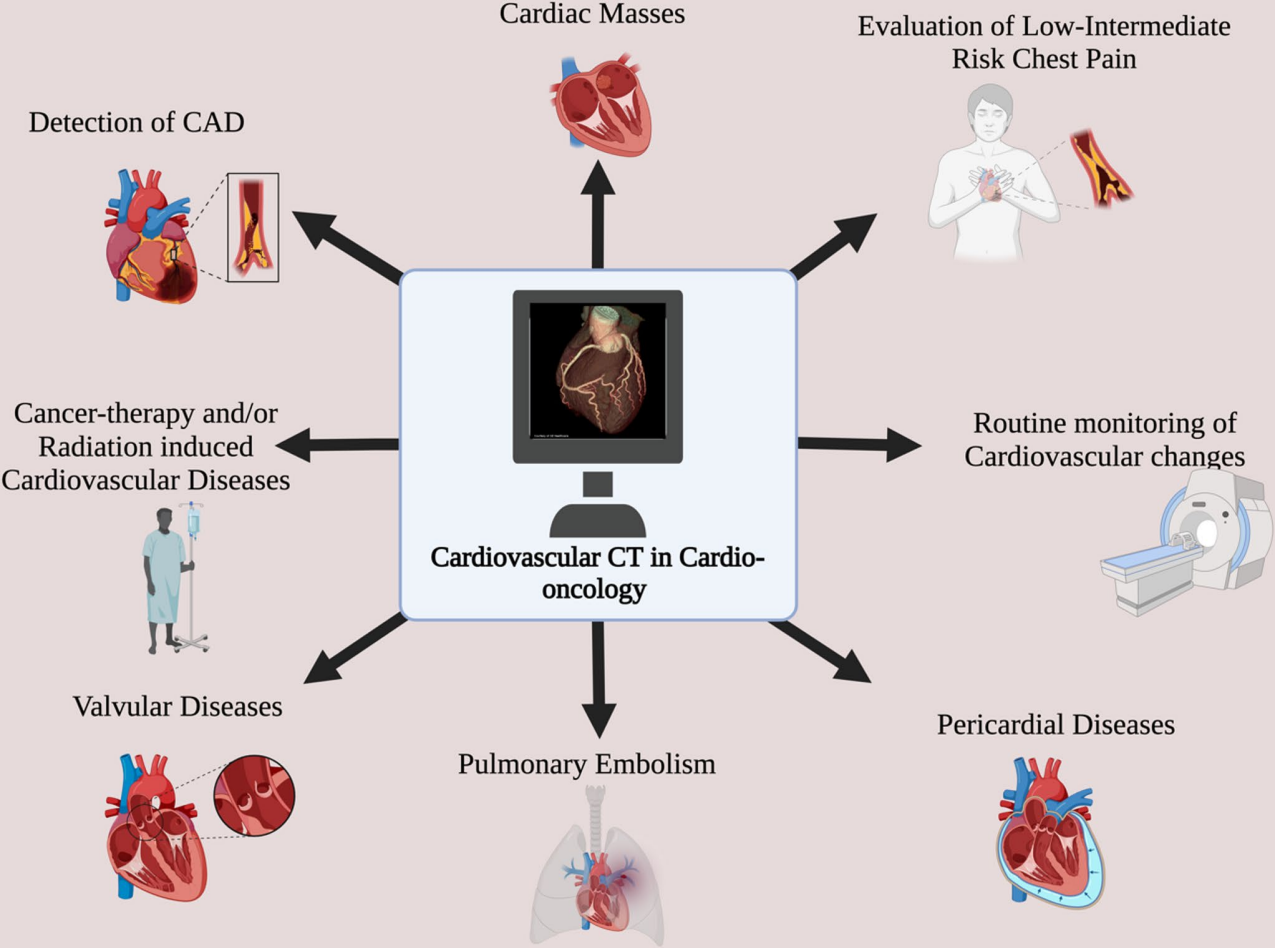
Cardiovascular CT Applications in Cardio-oncology. Created in BioRender. Erbay, M. (2023) BioRender.com/d50a424

Patients with cancer have an increased risk of CAD due to shared risk factors, radiation therapy, and chemotherapy-induced cardiotoxicity [7]. Using contrast, CCTA can identify obstructive and nonobstructive coronary artery disease, enabling early intervention, optimization of CV risk, and optimal medical or interventional management strategies (Table 2). This section discusses the utility of CCT in several cardiovascular disease states (Fig. 1).

**Table 2:** Utility of computed tomography (CT) in cardio-oncology

Utility of CCT in Cardio-Oncology
*Advantages*
Non-invasive and high-resolution imaging of the coronary arteries
Assessment of obstructive or nonobstructive CAD and CV risk optimization Optimal medical management of nonobstructive CAD
Radiation induced CVD
Preplanning for transcatheter procedures (TAVI, etc.) Valvular diseases Radiation sequalae in the heart and adjacent structures (lung, peripheral vasculature)
Detection of cardiomyopathy, pericardial disease and pulmonary embolism
*Limitations*
Risk of exposure to ionizing radiation
Potential risks of iodinated contrast
Artifacts (motion, calcium-blooming, cone beam, beam-hardening, banding)
Limited application in patients with certain conditions (iodinated contrast allergy, kidney disease)
*Current Indications*
Enhanced Assessment of CV risk using CAC scoring
Ruling out ACS in cancer related Takotsubo syndrome
Monitoring high-risk individuals for early primary intervention strategies

CCT: Cardiovascular computed tomography, CAD: coronary artery disease, CVD: cardiovascular disease, HF: heart failure, CV: cardiovascular, CAC: coronary artery calcium, ACS: acute coronary syndrome

### Coronary Artery Disease

In addition to its use in cancer diagnosis and tumor staging, CT can also be used for the detection of coronary atherosclerosis. CCT has emerged as a valuable tool to visualize coronary anatomy, including bypass grafts and stents, to detect coronary artery plaques, including plaque burden, degree of stenosis, calcification, and other characteristics. CCT particularly plays a crucial role in detecting subclinical atherosclerosis and non-obstructive/non-calcified plaque by quantifying CAC. Traditional clinical ASCVD risk scores do not take into account cancer-related risk factors such as specific chemoradiation therapies and the presence of somatic mutations defined as clonal hematopoiesis of indeterminate potential (CHIP), which has been associated with a higher degree of CVD in an older population without a history of cancer [8]. Readily available, routinely obtained non-cardiac chest CT for cancer evaluation can provide the chance to estimate the extent of CAC for ASCVD risk stratification, in addition to traditional ASCVD 10-year risk stratification [6, 9].

Non-cardiac CT scans can be integrated to measure CAC scores after necessary reconstructions such as evaluation with slice thickness of 2–3 mm [10]. CAC DRS (data reporting system) helps risk classification based on Agatston or visual CAC scores [11]. The Agatston score category identifies CAC = 0 as very low risk, CAC 1–99 as mildly increased, CAC 100–299 as moderately increased and CAC > 300 as moderately to severely increased risk (Table 3).

**Table 3:** CAC-DRS category risk classifications and treatment recommendations by SCCT expert consensus document [8]

CAC-DRS category risk classifications and treatment recommendations by SCCT expert consensus document		
**a. Agatston Score**		
**CAC Score**	**Risk**	**Treatment Recommendation**
CAC-DRS 0	0	Very low risk, statin generally not recommended*
CAC-DRS 1	1–99	Mildly increased risk, moderate intensity statin
CAC-DRS 2	100–299	Moderately increased risk, moderate to high intensity statin + ASA 81 mg
CAC-DRS 3	> 300	Moderately to severely increased risk, high intensity statin + ASA 81 mg
**b. Visual Score**		
**CAC Score**	**Risk**	**Treatment Recommendation**
CAC-DRS 0	0	Very low risk, statin not recommended*
CAC-DRS 1	1	Mildly increased risk, moderate intensity statin
CAC-DRS 2	2	Moderately increased risk, moderate to high intensity statin + ASA 81 mg
CAC-DRS 3	3	Moderately to severely increased risk, high intensity statin + ASA 81 mg

ASCVD risk assessment by risk models should also accompany medical decision-making with the use of CAC scoring

*Excluding familial hypercholesterolemia

CAC: Coronary Calcium Score, DRS: Data Report System, ASA: acetylsalicylic acid (aspirin)

(Data from: Lopez-Mattei J, et al. J Cardiovasc Comput Tomogr. 2023;17(1). 10.1016/j.jcct.2022.09.002, with permission from Elsevier) [8]

CAC is a robust predictor of CVD and increasing CAC scores are associated with higher all-cause mortality in patients with a history of cancer [12]. Enhancing traditional ASCVD risk estimations with CAC scores, CV risk groups can be reclassified to help control CV risk by implementing primary prevention strategies such as statins [4, 6].

In addition to these applications, CCTA is an effective tool with high negative predictive value to rule out obstructive CAD. This is particularly useful for patients with cancer, who often face physical constraints that limit their ability to undergo exercise-based stress testing and have increased risk of complications associated with invasive coronary angiography (i.e., increased risk of bleeding due to low platelet counts). In this population, CT - Fractional flow reserve (CT-FFR) enables a noninvasive assessment of hemodynamic significance of stenoses in the coronary arteries [13]. CT-FFR represents a relatively recent advancement that enhances the functional assessment capability of CCT, addressing its limitation of stenosis specificity [14].

CCT is also useful in identifying nonobstructive lesions and high-risk plaque features such as thin fibrous cap, lipid core in plaques with high risk of rupture and positive remodeling [15]. Numerous outcome studies provide a strong correlation between CAC scores and cardiovascular mortality. Patients with cancer with a higher CAC score are at a greater risk of CV events [16]. By identifying nonobstructive plaque characteristics, CCT assists in initiating appropriate primary/secondary preventive interventions such as statins, antiplatelet therapy, and/or, ezetimibe, proprotein convertase subtilisin/Kexin type 9 (PCSK9) inhibitors for optimal lipid-lowering strategies [6]. Implementing early interventions to mitigate CV risk profiles of patients with cancer may lead to improved outcomes and overall survival rates; however, current data is still lacking on outcomes of early interventions of ASCVD risk factor modification in the cancer population [6, 17–19]. Increased risk of CVD in people with cancer is a well-recognized complication that may arise early in cancer treatment or later during survivorship care; proactively monitoring these patients to lower CV risk at the earliest opportunity is of utmost importance [6, 20].

Some cardiotoxic agents can mimic acute coronary syndrome (ACS), complicating diagnosis and management. Mimicking ACS can happen through various mechanisms. For example, 5-fluorouracil (5-FU) can cause coronary vasospasm, immune checkpoint inhibitors (ICI) can cause myocarditis, trastuzumab cardiotoxicity can cause left bundle branch block, and cardiac dysfunction during treatment [21–23]. In these cases, CCTA plays a pivotal role in excluding or diagnosing ACS by providing detailed imaging of the coronary arteries. CCTA, in patients with a lower pretest probability of ASCVD, can help determine if invasive therapy is necessary, which is especially important for patients with a higher risk of procedural complications, such as those with hematologic abnormalities leading to increased bleeding risk.

### Valvular Diseases

Valvular dysfunction is more prevalent among patients with cancer and progresses during cancer treatment in more than 30% of the patients reported in several studies [24, 25]. CCT can assess valve morphology, annular size, severity of calcification, and/or severity of aortic or mitral valve stenosis. This is a critical tool in planning for surgical or percutaneous structural interventions, including transcatheter aortic/mitral interventions (TAVR / TMVR).^5,26^ Lastly, CCT can be used to evaluate aortic arch and/or ascending aorta calcification (i.e. porcelain aorta) which can arise from certain cancer treatments, such as mediastinal radiation, and can be associated with a higher risk of perioperative strokes during cardiac surgeries or transcatheter valvular procedures [27].

### Cardiomyopathies

Cancer treatment related cardiac dysfunction (CTRCD) is a serious and prevalent short and long term sequalae of cancer treatment that can cause both diastolic and systolic dysfunction. Young individuals with a history of cancer are at 15 times higher long-term risk of developing heart failure due to CTRCD [28]. Traditionally, CTRCD has been most closely associated with anthracyclines and anti-HER2 (human epidermal growth factor receptor 2) therapies [29–31]. CCT has an important role in ruling out CAD and ischemic causes of cardiomyopathy during the evaluation of new suspected CTRCD. There are emerging causes of treatment-related cardiomyopathy, including ICI-myocarditis. Particularly in the case of ICI myocarditis where troponin is elevated, cardiac dysfunction must be distinguished from ischemic cardiomyopathy [24]. CCT provides similar accurate left ventricular ejection fraction (LVEF) estimations compared to cardiac magnetic resonance imaging (cMRI) [32, 33]. With contemporary dose modulation acquisition techniques, CT-derived LVEF measurement can be conducted with very low-radiation doses [34]. The excellent spatial resolution in CCT enables visualization of coronary arteries and thus identifying cardiomyopathy etiology [35]. Nonetheless, guidelines recommend use of cMRI and echocardiography in measuring LVEF; CCT requires iodinated contrast and ionizing radiation, making it a less attractive option to assess cardiac function as a standalone indication [36]. cMRI is the preferred imaging modality for differentiating cardiomyopathies due to the additional soft tissue characterization, particularly the presence of fibrosis, and the lack of ionizing radiation and iodinated contrast required. It is important to recognize that in the functional assessment of heart failure, other imaging modalities such as echocardiography or cMRI are favored in the current guidelines [37].

### Pericardial Disease

CCT helps identify the heterogeneous spectrum of pericardial diseases, including pericardial effusions that may arise from active malignancy or a consequence of the cancer treatment, pericardial thickness, and pericardial calcifications from chronic pericardial inflammation and/or treatments (i.e. radiation) [38]. Readily available chest CT scans among patients with cancer can also raise a suspicion of pericardial diseases, especially pericardial effusion. [39] While cMRI offers high-resolution imaging of pericardial and cardiac anatomy, Hounsfield unit (HU) measurement in CCT can be helpful in the discrimination of exudative or transudative effusion. Exudative effusions yield a higher HU due to higher content of pericardial fluid albumin, lactate dehydrogenase (LDH), and white blood cells [40]. The high resolution of CCT, compared to transthoracic echocardiography (TTE) or cMRI, can be necessary to evaluate the thin pericardium and particularly to detect the presence of calcification in constrictive pericarditis.

### Radiation-Induced Cardiovascular Diseases

Radiation-induced cardiovascular disease (RI-CVD) refers to any cardiovascular compromise in patients receiving radiation therapy [26]. Chest radiation therapy is a cornerstone of treatment for certain cancers including lung and breast cancers. RI-CVD leads to multiple cardiovascular complications that can be seen on a single CCT, such as coronary artery disease, valvular dysfunction, myocardial dysfunction, cardiomyopathies, and pericardial [41, 42]. CCT provides detailed information about cardiac anatomy, coronary arteries, valves, pericardium, and extracardiac cardiac structures. Valvular dysfunction, particularly of the aortic and mitral valves, occurs due to accelerated valvular calcification and can be well seen using CCT imaging. CCT, as explained above, is useful in the assessment of obstructive or nonobstructive CAD [6, 41]. Furthermore, CCT may help radiation oncologists to accurately define cancerous target volume and spare critical cardiac structures, minimizing the risk of cardiotoxicity while optimizing tumor control [43].

### Pulmonary Embolism

PE is a potentially life-threatening condition that can occur more prevalently during cancer treatment. CCT involves thoracic imaging which visualizes pulmonary arteries and thus incidentally, if the contrast bolus is appropriately timed, CCT can diagnose or rule out pulmonary embolism (PE) [44]. Furthermore, it can be used to assess hemodynamic consequences of PE, including right heart strain by evaluating the relative sizes of the right and left ventricles. To utilize CCTA for PE evaluation, special consideration must be paid to the timing of contrast bolus to ensure full opacification of both the coronary arteries and the pulmonary arteries.

### Cardiac Masses

Cardiac masses encompass various entities such as thrombi, vegetations, benign tumors like myxomas and papillary fibroelastomas, as well as rare malignant primary or metastatic tumors. CCT surpasses cMRI with its high spatial resolution. CCT can assess for tumor vascularity using contrast enhancement, calcification extent, the presence of adipose tissue, and simultaneous extracardiac cancer staging. Particularly for masses adjacent to prosthetic valves, CCT is the preferred choice and over cMRI in detecting calcified masses [45]. Additionally, CT’s enhanced spatial resolution aids in 3D reconstruction, and may assist in radiation treatment planning for metastatic or primary malignancies involving the heart [46]. However, due to its ability to distinguish tissue characteristics, cMRI is the preferred modality for distinguishing cardiac thrombus from malignancies, and for detailed characterization of cardiac tumor types [47].

### Preplanning for Transcatheter Procedures

CCT has an important role in preplanning for transcatheter procedures, particularly for TAVR. CCT encompasses a comprehensive, noninvasive evaluation of the sequelae of radiation exposure in the heart and adjacent structures such as the lungs and peripheral vasculature. The holistic view of the aortic root and valvular anatomy is crucial and appropriate for patient selection as emphasized by SCCT Expert Consensus statement [48].

## Advantages of Cardiovascular CT Compared to Other Imaging Modalities

CCT offers several advantages over other imaging techniques in cardio-oncology. CCT provides superior spatial resolution and the ability to evaluate the entire coronary tree and extracardiac structures. In comparison to cMRI, CCT is less susceptible to motion artifacts, making it more suitable for patients who have difficulty remaining still during the imaging process [10, 11, 49]. Additionally, CT imaging is faster and more readily available than MRI, which can be important in the timely evaluation of cardio-oncology patients. Lastly, CCT plays an important role in the assessment of cardiac masses, with the ability to also evaluate calcified elements, within the heart as mentioned above [45].

**Fig. 2:**
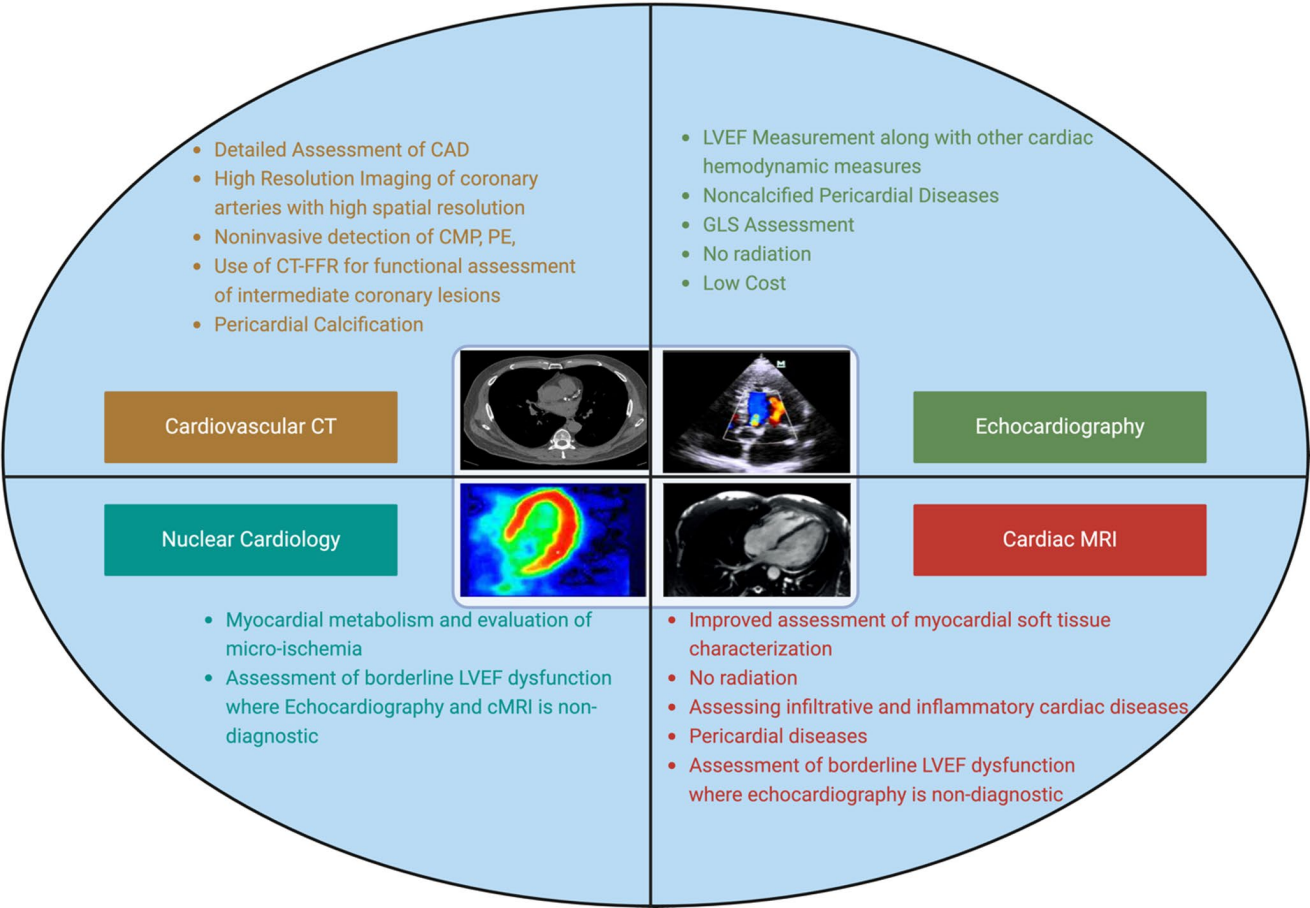
Multimodality Imaging in Cardio-oncology. CAD: Coronary Artery Disease, LVEF: Left Ventricular Ejection Fraction, GLS: Global Longitudinal Strain, CT-FFR: Fractional Flow Reserve– Computed Tomography, CMP: cardiomyopathy, PE: Pulmonary Embolism. Created in BioRender. Erbay, M. (2023) BioRender.com/a27k779

Overall, CCT combines excellent spatial resolution, comprehensive cardiac assessment, and accessibility, making it a valuable imaging modality in cardio-oncology for the detection, monitoring, and management of cardiovascular complications associated with cancer and its treatments (Fig. 2).

## Limitations of Cardiovascular CT in Cardio-Oncology

While CCT offers numerous advantages in cardio-oncology, it is essential to acknowledge its limitations. Understanding these limitations is crucial for healthcare providers to make informed decisions regarding the appropriate use of CCT in cardio-oncology patients.

### Risk of Ionizing Radiation

CCT involves the use of ionizing radiation, which poses a potential risk to patients who often require repeated imaging studies for staging during their cancer treatment course [50]. It is important to balance the potential benefits of CCT with the radiation risk, especially in younger patients with breast cancer, those undergoing radiotherapy, and those with genetic predispositions to developing malignancies. Radiation exposure should be decreased with the As Low As Reasonably Achievable (ALARA) approach which constitutes three components: lowering time, maximizing the distance, and the appropriate shielding [51]. Although retrospectively gated heliacal CCT has a high amount of radiation exposure as high as 9–32 mSv, several dose reduction strategies have been developed such as ECG-correlated tube current modulation resulting in 37% radiation dose reduction in CCT [52]. Additionally, prospective axial gating protocol offers up to 77% reduction in radiation dose. Thus, the modern CCT procedure typically results in low amounts of radiation, outweighing its risks (Table 4). Because prospective gating does not capture during systole, CCT may have limited application for serial monitoring of LVEF due to the risk of radiation [53]. Radiation dose reduction strategies, including appropriate patient selection and optimization of scanning protocols, should be employed to minimize radiation exposure while maintaining diagnostic image quality [54].

**Table 4:** Radiation doses from different heart CT protocols and other alternative imaging modalities. 1 mSv is equal to the average accumulated background radiation dose to an individual for 1 year in the United States [57]

Imaging Technique	Effective Radiation Doses - millisievert (mSv)
CCTA	1.3–9 mSv*
Calcium score	1.7 mSv
NCCT	5.1 mSv
Chest X ray	0.1 mSv
Chest CT	6.1 mSv
SPECT/CT** [55]	7.7mSv
PET/CT*** [56]	8–25 mSv

*Radiation doses depend on the protocol used (retrospective/ prospective ECG gating)

** Myocardium only

*** Varies highly on body weight and amount of radiotracer injected

CCTA: Coronary computed tomography angiography, NCCT: Non-contrast computed tomography

### Use of Iodinated Contrast

CCT commonly requires the use of iodinated contrast agents, which can pose risks for patients with impaired kidney function [58]. Contrast-induced nephropathy (CIN) is defined as an elevation of serum creatinine of more than 25% or ≥ 0.5 mg/dl (44 µmol/l) from baseline within 48 h of exposure. Also, even though rare, patients with a known allergy to iodinated contrast agents may not be suitable candidates for CCT, and alternative imaging modalities or contrast agent protocols may need to be considered [59].

### Artifacts

Motion artifacts can significantly impact the accuracy and reliability of CCT images, leading to high false positive rates and potential diagnostic uncertainty [60, 61]. Techniques such as breath-holding instructions and heart rate control, with the administration of medications such as beta-blockers when needed, can help mitigate motion artifacts; however, challenges may persist, especially in patients who struggle with breath-holding or have an irregular heart rate. Blooming artifacts arise due to high-density structures, such as calcium or stents, making them appear larger than their actual size. This can be due to partial volume averaging, motion, or beam hardening [62]. Blooming artifacts can compromise the accuracy of CT images, leading to difficulties in accurately assessing nearby anatomical structures and potentially leading to false-positive findings [62].

Beam hardening artifacts can compromise CCT images by creating shadings mimicking myocardial ischemia. Cone-beam artifact occurs when the cone-beam geometry is inappropriate, shadings occur near the spine and ribs. Banding artifacts caused by irregular heartbeats or suboptimal gating scheme can lead to non-diagnostic images. β-blocker use can reduce heart rate variation and more robust gating schemes can solve these issues [63].

### Limited Application in Patients with Certain Conditions

While functional assessment of intermediate coronary stenosis is enhanced with the addition of FFR, alternative imaging modalities such as MRI, nuclear, and echocardiography stress imaging offer a more comprehensive evaluation of cardiac function and ischemia and should be considered when CCTA will likely not be diagnostic. Alternative imaging options, such as cMRI, should be explored in these situations to ensure patient safety and diagnostic accuracy. Additionally, CT has limited soft tissue contrast and evaluation of some diagnoses may be better suited to echocardiography or cMRI, including infiltrative cardiomyopathies, fibrosis, or myocardial edema. Lastly, a major limitation of CCTA is the need to have a controlled heart rate for optimal imaging, usually a heart rate < 60 bpm, which often requires administration of B-blockers. Additionally, nitrates are required for standard clinical CCTA exams to allow for accurate assessment of coronary stenoses. Given that patients with cancer often have sinus tachycardia and borderline low blood pressure, there may be clinical limitations to obtaining CCTA in some circumstances [64, 65].

## Future Directions in the Use of Cardiovascular CT in Cardio-Oncology

### Integration of Artificial Intelligence Algorithms

As technology and research continue to advance, there are promising future directions for the use of CCT in cardio-oncology. The integration of artificial intelligence (AI); machine learning (ML) and deep learning (DL) algorithms into CCT analysis holds the potential for automated image interpretation, improved precision, personalized care, and enhanced ASCVD risk stratification in cardio-oncology patients [66, 67]. Current ML algorithms can accurately predict the stenoses grade and ischemia as shown in a CT-FFR study [67]. In this study, an ML algorithm was trained on 581 vessels from the prospective PACIFIC trial to develop an ML score for ischemia prediction. The ML score was then applied to predict myocardial blood flow from corresponding cardiac PET scans and ML score performance was compared with CCTA reads and noninvasive CT-FFR. The study showed that ML algorithm have a higher area under the receiver-operating characteristic curve (AUC) compared to FFR-defined ischemia and impaired blood flow prediction. A study from CAC Consortium developed an ML model including 77 variables and is trained with data from 66,636 asymptomatic subjects. The model is evaluated using a cross-validation framework from the available data and predictive value of the proposed model is compared to ASCVD and CAC scores based on their performance in AUC [68]. AUC in CVD and coronary heart disease (CHD) death prediction were superior to ASCVD and CAC scores. [CVD prediction: 0.845 (ML) 0.821 (ASCVD) 0.781 (CAC) / CHD prediction: 0.86 (ML) / 0.835(ASCVD) 0.816 (CAC); *p* < 0.0001 for all].

Deep learning (DL) is a subset of ML that uses neural networks with multiple hidden layers for capturing complex patterns and image recognition. It’s primarily used for large datasets and focuses on deeper interactions. Several studies using DL algorithms that are externally validated, meaning that is validated by a different cohort than its training cohort for minimizing the overfitting and maximizing generalizability, have reported that automated CAC score prediction is noninferior to expert-annotated CAC scores [69, 70]. Another study highlights the use of DL algorithm on non-ECG gated chest CTs to detect incidental CAC > 100, as this score is associated with a worse CVD and mortality outcomes independent from traditional risk factors [71].

Thus, DL and ML algorithms are promising tools to allow for opportunities for earlier intervention and CVD prevention. Future work in the field of preventive cardiology should focus on supporting implementation of AI algorithms, identifying subclinical CVD in patients with a history of cancer and further personalizing the CVD prevention in people with cancer [12, 34, 64–67, 51].

## Conclusion

In conclusion, CCT plays a role in risk stratification through the detection of coronary artery disease in both cardiac and non-cardiac scans as a pivotal step in preventive cardiovascular event management. By accurately assessing CAD risk, clinicians can implement tailored preventive measures, further reducing the incidence of cardiovascular events. Moreover, CCTA is an invaluable imaging modality for patents presenting with CAD symptoms, whether stable or acute. In evaluation of cardiomyopathy, CCT aids in distinguishing between ischemic cardiomyopathy or chemotherapy related cardiotoxicity. The role of CCT extends beyond CAD assessment, encompassing the evaluation of valves, pericardium, and cardiac masses, offering a holistic perspective on cardiac health and contributing to informed clinical decision-making. As advancements in cancer treatment leads to an increasing number of cancer survivors, CCT can be an invaluable tool in providing information on cardiac anatomy including the presence of preexisting or acquired cardiovascular disease through the continuum of the patient’s cancer journey.

## Key References

- Lopez-Mattei J, Yang EH, Baldassarre LA, et al. Cardiac computed tomographic imaging in cardio-oncology: An expert consensus document of the Society of Cardiovascular Computed Tomography (SCCT). Endorsed by the International Cardio-Oncology Society (ICOS). *J Cardiovasc Comput Tomogr*. 2023;17(1). 10.1016/j.jcct.2022.09.002.
-
  - This paper includes several recommendations for use of CCT in cardio-oncology population.
- Baldassarre LA, Ganatra S, Lopez-Mattei J, et al. Advances in Multimodality Imaging in Cardio-Oncology: JACC State-of-the-Art Review. *J Am Coll Cardiol*. 2022;80(16). 10.1016/j.jacc.2022.08.743.
-
  - This review provides valuable and holistic approach to cardiovascular imaging in populations of patients with cancer.
- Miller RJH, Mamas MA, Tamarappoo B, et al. Extensive coronary artery calcification is associated with all-cause mortality patients with a history of cancer. *J Cardiovasc Comput Tomogr*. Published online 2023. 10.1016/j.jcct.2023.04.001.
-
  - This review explores that higher coronary artery calcification increases the all-cause mortality in patients with a history of cancer. Using nongated scans, CAC scanning can be used for risk stratification in this population.

## Data Availability

No datasets were generated or analysed during the current study.

## Abbreviations

CCT: Coronary Computed Tomography
CVD: Cardiovascular Disease
ASCVD: Atherosclerotic Cardiovascular Disease
CT: Computed Tomography
CAC: Coronary Artery Calcium
CCTA: Coronary Computed Tomography Angiography
ACS: Acute Coronary Syndrome
LOE: Level of Evidence
CT-FFR: Computed Tomography Fractional Flow Reserve
CHIP: Clonal Hematopoiesis of Indeterminate Potential
LVEF: Left Ventricular Ejection Fraction
cMRI: Cardiac Magnetic Resonance Imaging
CTRCD: Cancer Therapy-Related Cardiac Dysfunction
AI: Artificial Intelligence
